# Gliomas and the vascular fragility of the blood brain barrier

**DOI:** 10.3389/fncel.2014.00418

**Published:** 2014-12-12

**Authors:** Luiz Gustavo Dubois, Loraine Campanati, Cassia Righy, Isabella D’Andrea-Meira, Tania Cristina Leite de Sampaio e Spohr, Isabel Porto-Carreiro, Claudia Maria Pereira, Joana Balça-Silva, Suzana Assad Kahn, Marcos F. DosSantos, Marcela de Almeida Rabello Oliveira, Adriana Ximenes-da-Silva, Maria Celeste Lopes, Eduardo Faveret, Emerson Leandro Gasparetto, Vivaldo Moura-Neto

**Affiliations:** ^1^Instituto Estadual do Cérebro Paulo Niemeyer, Rua do RezendeRio de Janeiro, Brazil; ^2^Laboratório de Morfogênese Celular, Instituto de Ciências Biomédicas da, Universidade Federal do Rio de JaneiroRio de Janeiro, Brazil; ^3^Programa de Pós-Graduação em Odontologia, Escola de Ciências da Saúde (ECS), Universidade do Grande Rio (UNIGRANRIO)Duque de Caxias, Brazil; ^4^Centro de Neurociência e Biologia Celular, Faculdade de Medicina, Universidade de CoimbraCoimbra, Portugal; ^5^Instituto de Ciências Biológicas e da Saúde, Universidade Federal de Alagoas, MaceióAlagoas, Brazil

**Keywords:** glioblastoma, blood-brain barrier, miRNA, exosomes, neural stem cells, tumor-related epileptic seizures, brain tumor related edema

## Abstract

Astrocytes, members of the glial family, interact through the exchange of soluble factors or by directly contacting neurons and other brain cells, such as microglia and endothelial cells. Astrocytic projections interact with vessels and act as additional elements of the Blood Brain Barrier (BBB). By mechanisms not fully understood, astrocytes can undergo oncogenic transformation and give rise to gliomas. The tumors take advantage of the BBB to ensure survival and continuous growth. A glioma can develop into a very aggressive tumor, the glioblastoma (GBM), characterized by a highly heterogeneous cell population (including tumor stem cells), extensive proliferation and migration. Nevertheless, gliomas can also give rise to slow growing tumors and in both cases, the afflux of blood, via BBB is crucial. Glioma cells migrate to different regions of the brain guided by the extension of blood vessels, colonizing the healthy adjacent tissue. In the clinical context, GBM can lead to tumor-derived seizures, which represent a challenge to patients and clinicians, since drugs used for its treatment must be able to cross the BBB. Uncontrolled and fast growth also leads to the disruption of the chimeric and fragile vessels in the tumor mass resulting in peritumoral edema. Although hormonal therapy is currently used to control the edema, it is not always efficient. In this review we comment the points cited above, considering the importance of the BBB and the concerns that arise when this barrier is affected.

## The blood-brain barrier

It has been one hundred years since Edwin E. Goldmann discovered the blood-brain barrier (BBB). Using trypan-blue dye intravenous injections in several animals, he observed that it spread throughout the body, except for the brain and spinal cord, which remained unstained. When trypan blue was injected into the subarachnoid space at the lumbar level, or in the cisterna magna in young rabbits, the Central Nervous System (CNS), including the choroid plexus (CP), were stained (Goldmann, 1909; Bentivoglio and Kristensson, 2014), confirming the presence of a previously unknown barrier between the blood and CNS structures.

## Choroid plexus

The CP is a structure present in the CNS that also functions as a tissue barrier. It is responsible for the synthesis of the cerebrospinal fluid (CSF) and its major proteins, metabolites and a diversity of other molecules. Anatomically, the CP is a group of thin membranes located inside the lateral, third and fourth ventricles. It is basically composed of a monolayer of specialized epithelial cells derived from the ependyma that cover the ventricles walls. This monolayer coats a highly perfused stroma containing permeable fenestrated blood vessels, fibroblasts, dendritic cells and macrophages (Redzic et al., 2005). Its degree of vascularization exceeds in 10 times those of the brain parenchyma (Keep and Jones, 1990).

One of the best-described CP-secreted molecules is insulin-growth factor 2 (IGF2), which promotes neuronal proliferation and survival in the developing mouse cortex (Lehtinen et al., 2011). The presence of tight junctions along the basolateral membrane limits the exchange between the CSF and the CP connective tissue, preventing the passage of blood-derived cells and proteins (Vorbrodt and Dobrogowska, 2003). Tight junctions, combined with the expression of several transmembrane transporters, make CP cells the effectors of the blood-CP-CSF barrier (Spector, 2010). The CP is also responsible for the synthesis and generation of nearly two-thirds of the CSF total volume via the secretion of water, ions and macromolecules (Johanson et al., 2008). Aquaporins (AQPs) play a pivotal role in controlling water circulation, particularly AQP-1, which is present in the membrane of the CP cells (Benga et al., 1986).

## Astrocytes

**Figure 1:**
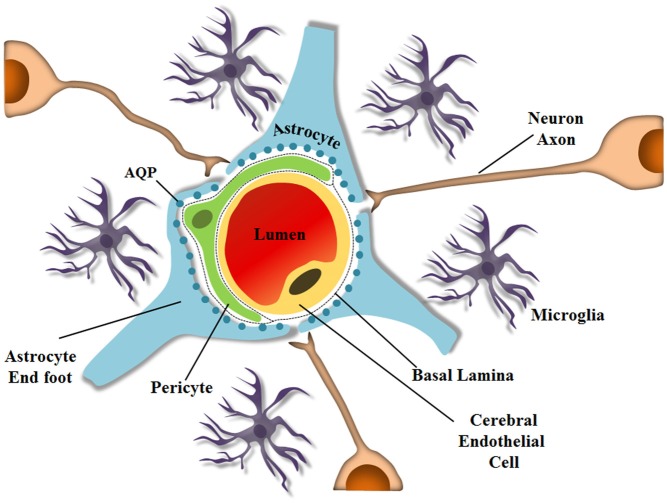
**Schematic representation of the neurovascular unit at the capillary level**. The BBB is composed of several cell types and ECM molecules in close association. Highly specialized and polarized endothelial cells, basal lamina, pericytes, and astrocyte endfeet, which by wrapping the microvessel walls, establish communication with neurons in the neurovascular unit. The neurovascular unit is important to maintain optimal brain function. Pericytes and astrocytes are important in barrier induction and maintenance. Microglia are CNS-resident immune cells. (Diagram by de Spohr, TCLS). Adapted from Abbott (2013).

Astrocytes also express AQPs, especially on their endfeet, which are closely associated with endothelial cells that are also part of the BBB. Astrocytes contribute to the selection and exchange of molecules through the barrier, and although a hundred years have passed since the BBB was discovered, the mechanisms by which some drugs or parasites enter the brain still remain unsolved. The BBB is a specialized non-permeable barrier in cerebral microvessels. It consists of endothelial cells united by tight junctions, astrocytic endfeet surrounding blood vessels, pericytes embedded in the vessel basement membranes (BMs), microglia and neurons, all playing essential roles in CNS homeostasis (Abbott et al., 2010; Aryal et al., 2014), as shown schematically in Figure 1. Together, the components important for BBB integrity and maintenance, including the extracellular matrix, are known as the neurovascular unit, which forms a highly coordinated system that dynamically regulates the cerebral microvascular permeability (Bicker et al., 2014).

Astrocytes are involved in several other processes important for normal brain development, such as neurogenesis, myelination, synapse formation, neuronal migration, proliferation, differentiation, neuronal signaling, production of neurotrophic factors, formation of scar tissue after neuronal loss, immune activation, inflammation, and BBB integrity (Anton et al., 1997; Pfrieger and Barres, 1997; Gomes et al., 1999; Lim and Alvarez-Buylla, 1999; de Sampaio e Spohr et al., 2002; Rouach et al., 2002; Kielian, 2008; Diniz et al., 2012; Herculano-Houzel, 2014). Astrocytic endfeet protrusions interact with microvascular endothelial cells by covering the capillaries forming the *glia limitans*. A single astrocyte may contact multiple capillaries, and this interaction is required for endothelial cells to acquire properties of the BBB (Hayashi et al., 1997; Abbott, 2002; Abbott et al., 2006; Obermeier et al., 2013). Interestingly, astrocytic coverage of vessel endothelial cells and pericytes is discontinuous at only a few sites, and in these rare situations microglial processes can be observed touching the basal lamina (Lassmann et al., 1991; Mathiisen et al., 2010). Gaps in the endfeet coverage of pericytes occur more frequently, allowing neuropil elements to contact the basal lamina (Mathiisen et al., 2010). These astrocytes also interpose in the contacts between microvessels and neurons, important for coordinating oxygen and glucose transport for neural activity through regulation of local blood flow (Zonta et al., 2003; Iadecola, 2004; Takano et al., 2006; Iadecola and Nedergaard, 2007).

Astrocytes control BBB tightness by secreting soluble factors that influence endothelial cells. Additionally, the presence of numerous astrocyte endfeet close to the BBB facilitates the rapid regulation of BBB permeability, which is important for the innate immune system in the brain (Prat et al., 2001; Gimsa et al., 2013). Astrocytes participate in the formation of the BBB by inducing tight junction formation in endothelial cells, by modulating the expression and polarization of transporters, and by promoting specialized enzyme systems (DeBault and Cancilla, 1980; Janzer and Raff, 1987; Abbott, 2002; Lee et al., 2003; Haseloff et al., 2005; Abbott et al., 2006). Astrocytes also up regulate the expression of ZO-1 and occludin and modulate the redistribution of CD31, claudin-5 and ZO-1 from a diffuse pattern to the cell borders in endothelial cells (Siddharthan et al., 2007; Colgan et al., 2008; Al Ahmad et al., 2011). The interaction between astrocytes and endothelial cells is a “two-way street”, since endothelial cells also stimulate astrocyte growth and differentiation (Mi et al., 2001; Abbott et al., 2006).

Astrocytes induce and maintain barrier properties in endothelial cells postnatally, while during embryonic development these properties are first regulated by interactions with neural progenitors, and then by pericytes, since in many species, astrocyte differentiation occurs close to birth (Daneman et al., 2010). It is possible that astrocyte precursors may determine the fate of cerebral vascular endothelial cells in the immature neural environment by releasing soluble factors, as proposed based on *in vitro* co-culture experiments by Hayashi et al. (1997). Clearly, astrocytes have many features that are important to BBB physiology. The situation in the brain becomes complex when these astrocytes become malignant, as in astrocytoma. Intense neovascularization occurs in the brain around the tumor region, to contribute to tumor growth. This vascularization originates in endothelial cells, but the vessels are also constituted by the tumor cells adapted to this new function.

## BBB and glioma

Gliomas comprise a group of tumors that originate in the brain. They form a special group of neoplasias for which no cure is currently available, and only modest progress has been made in understanding their biology. Their specific denomination derives from the normal stromal cells of the brain—the macroglial cells—with which they share morphological traits and molecular markers, i.e., astrocytes (astrocytomas), oligodendrocytes (oligodendrogliomas) and ependymal cells (ependymomas) (Louis et al., 2007). In adults, gliomas account for 29% of all brain tumors; 80% of malignant primary brain tumors occurring in patients 65–84 years of age (see Figure 3; Dolecek et al., 2012). Gliomas also affect children and the most common pediatric histological types are astrocytomas (52%), primitive neuroectodermal tumors (PNETs), medulloblastomas (21%) and high-grade gliomas (19%) (Hemmati et al., 2003). Gliomas are highly heterogeneous, infiltrative and diffuse, with different degrees of invasiveness (Alves et al., 2011b). They can penetrate through the brain, colonizing the entire organ, sending their invasive cells far beyond the principal tumor mass. Despite this considerable invasive ability, gliomas rarely leave the nervous tissue to colonize other organs, remaining confined in the skull, with only little evidence of systemic spread (Louis et al., 2007).

**Figure 2:**
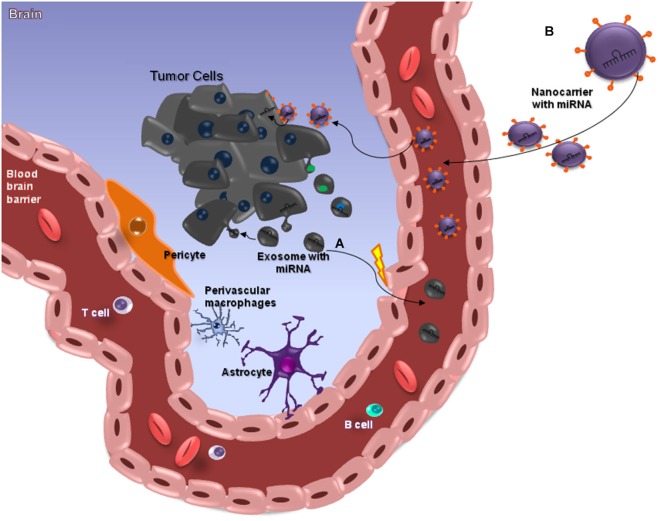
**miRNAs and the Blood-Brain Barrier**. MicroRNAs (miRNAs) are small (≈22 nt) regulatory and non-codified RNAs that are frequently deregulated in cancer and have shown promise as tissue-based markers for cancer classification and prognosis. They have emerged as key regulators of several pathogenic responses, although their role is largely unknown. The clinical use of siRNA-based therapies is highly promising, although dependent on the development of nanocarriers with appropriate features for systemic administration, since these small RNAs have a unstable structure. Nanocarriers protect the RNA from nuclease degradation and promote effective regulation of target genes, especially in brain tumors such as glioblastomas (GBMs) that are somehow protected from chemotherapeutic drugs by the blood–brain barrier (BBB), which is meant to protect the brain from noxious agents. Several strategies have been employed to deliver drugs across this barrier, and some of these can cause structural damage to the BBB. The ideal method for transporting drugs across the BBB would be controllable and not damage the barrier. Among the various presently available approaches, nanobiotechnology-based delivery methods are the most promising **(A)**. This image attempts to clarify the action of miRNAs in brain-cancer cells, through nanocarriers capable of crossing through the BBB. Exosomes are a class of secreted nanoparticles that have the ability to carry RNA and proteins, and therefore to be very important mediators of intercellular communication. The altered characteristics of exosomes in many diseases, such as cancer, suggests that they could be important for both diagnostic and therapeutic purposes, for instance as drug-delivery vehicles, especially for gene therapy as emphasized in this review **(B)**.

**Figure 3:**
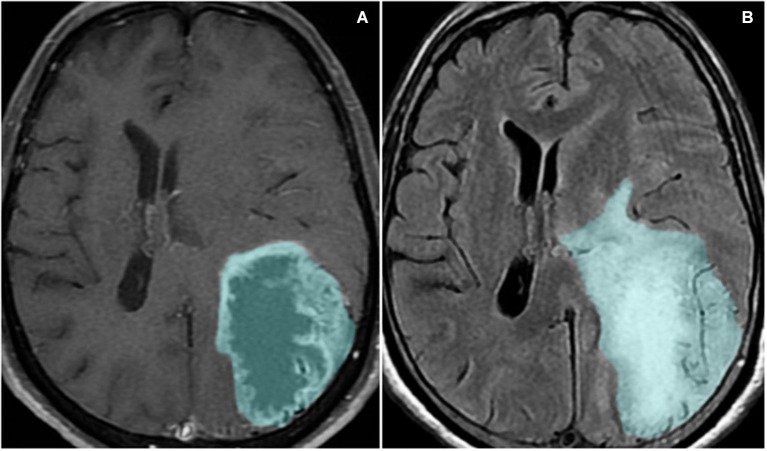
**Magnetic resonance imaging sequences T1-weighted with contrast (A) and FLAIR (B) show a left parieto-occipital mass with high signal on FLAIR and a surrounding area of vasogenic edema/tumor infiltration**. The lesion presents peripheral heterogeneous contrast enhancement, representing the disruption of the blood-brain barrier.

A universally accepted system for the classification of brain tumors was developed by the World Health Organization (WHO). This classification is intended to improve the accuracy of prognosis, and thus the effectiveness of therapeutic management of patients. Gliomas classification has long been based solely on the histopathological features of the tumor tissues, obtained through neurosurgical resections, which are whenever possible, the first line of therapeutic intervention. The diagnostic criteria include the morphology of the tumor cells, tissue architecture, and the immunohistological marker profiles (Louis et al., 2007).

The classification also includes a grading system that distinguishes four different grades of tumors: grades I, II, III and IV for astrocytomas and II or III for oligodendrogliomas and oligoastrocytomas. Lower-grade tumors (grades I and II) tend to be well differentiated and have few cellular anomalies or atypias, but in general, they closely resemble their non-neoplastic cellular counterparts. These tumors contain specific genetic alterations (Riemenschneider and Reifenberger, 2009) and evidence has been provided for the existence of progressive accumulation of additional genetic alterations. This is followed by the evolution of the tumor to higher grades, i.e., higher malignancy and progression rates (Louis, 2006).

Higher-grade tumors (grades III and IV) are anaplastic, showing signs of increased vessel density, cellular atypias, high mitotic activity and elevated cell density. Grade IV astrocytoma, better known under the name of glioblastoma (GBM) multiformis, or simply GBM, is the most common and aggressive form of glioma. Glioblastoma samples are characterized by a very high cell density, numerous atypias, areas of necrosis, and robust neoangiogenesis. Two forms of GBM have been identified: *de novo* GBM, the most frequent, and secondary GBM, which results from the evolution of a preceding low-grade astrocytoma (Louis et al., 2007). Glioblastoma accounts for 54% of all glioma subtypes (Dolecek et al., 2012). They show a highly aggressive course, poor treatment response, are incurable and patients have short-term survival expectancies (Miller and Perry, 2007).

Glioblastoma growth is closely associated with the formation of new vessels and one of the most serious clinical complication is the development of vasogenic brain edema, which dramatically increases the intracranial pressure (ICP) by BBB leakage (Noell et al., 2012). Glioblastomas are the most vascularized tumors in humans (Takano et al., 2010) and malignancy grade is directly related to endothelial proliferation (Daumas-Duport et al., 1988). Microvessel density is a standard indicator for the prognosis of patients with GBM. However, vessel formation is highly defective, resulting in vessels with abnormal morphology and function. Histologycal samples show vessels with variable diameters and permeability, heterogeneous distribution and irregular basal lamina (Dvorak, 2003).

One of the most widely accepted arguments to explain inefficient vessel formation in GBM is the relatively high amount of vascular endothelial growth factor (VEGF) present in the tumor mass (Bergers and Benjamin, 2003). Vascular endothelial growth factor is one of the most studied molecules in angiogenesis and acts mainly as a hypoxia-inducible factor; the areas expressing highest levels of VEGF are the regions of the necrotic core (Shweiki et al., 1992). Generally, VEGF is overexpressed in GBM and is responsible for the crosstalk between the tumor and the endothelial cells in order to promote angiogenesis.

Besides VEGF over expression, altered AQP expression in components of the BBB (astrocytic endfeet) has been correlated to glioma progression. Aquaporins are a family of integral membrane transport proteins that facilitate water efflux across cell membranes in response to osmotic gradients. So far, 14 AQPs have been characterized in humans and rodents (Papadopoulos and Verkman, 2013). In gliomas, AQP1 is upregulated *in vitro* in response to increased glucose consumption and glycolysis (Hayashi et al., 2007). The expression of AQP8 in human astrocytomas is associated with the pathological grade, being directly related to the aggressiveness of the tumor (Zhu et al., 2013). AQP9, on the other hand, is highly expressed in tumor stem cells, which are found in the tumor mass and are resistant to most available treatments (McCoy and Sontheimer, 2007).

Aquaporins have been described as key regulators of the BBB integrity. The AQP4 arrangement through the membrane contributes to edema resolution and it is believed that the upregulation of AQP4 observed in gliomas *in vivo* is a mechanism of compensation for the loss of the endfeet and the increase in the perivascular space (Noell et al., 2012) observed in gliomas.

In the early stages of glioma development there is no apparent disruption of the BBB; tumor own vasculature has not yet been formed and the tumor mass is sustained by normal brain vessels. As glioma progresses and aggravates, endothelial cells derived from normal vessels are roughly separated from the vessel main structure and form new angiogenic spots associated with the tumor site. As these cells must migrate when forming new vessels, they disrupt normal vessel structure to arrive at the tumor site. Because tumors secrete many different molecules that alter the normal microenvironment, endothelial cell migration is impaired, and this impairment is reflected in the vascular architecture. In GBM, the morphological alterations of blood vessels involve the formation of fenestrations and tight junctions disruption. Besides, the thickness of the basal lamina is altered and perivascular space is increased as well as the number of pericytes associated to the vessels (Hirano and Matsui, 1975; Bertossi et al., 1997).

The disruption of the BBB can be detected through magnetic resonance imaging (MRI), using a contrast medium (CM; Sage and Wilson, 1994). The standard CM used for GBM diagnosis in MRI is gadolinium, which under normal conditions is not able to cross the intact BBB. In the case of GBM, since there is a disruption of the blood brain barrier, gadolinium can diffuse into the tissue and characteristic ring enhancing lesions are often seen.

## Neural progenitor cells cross the BBB and decrease tumor size

It is currently accepted that GBMs can also be derived from tumor stem cells. As said before, GBMs are heterogeneous, due to the presence of non-tumor cells such as astrocytes, microglial cells and endothelial cells (Fonseca et al., 2012; Kahn et al., 2012; Lima et al., 2012). Endogenous, non-tumoral, neural stem cells are able to migrate from the subventricular zone (SVZ) toward glial brain tumors, damaged or regenerating tissue, and inflammation sites (Aboody et al., 2000; Tang et al., 2003; Synowitz et al., 2006; Walzlein et al., 2008; Díaz-Coránguez et al., 2013). Through xenotransplantation of GBM cells and experimentally induced tumors, it was shown that the number of neuroprogenitor cells (NPCs) attracted to the tumor bed decreases with age, and that this correlates with rapid tumor growth (Maslov et al., 2004; Synowitz et al., 2006; Walzlein et al., 2008). Neuroprogenitor cells of young and old mice have been shown to exhibit antitumorigenic effects by inducing GBM cell death (Glass et al., 2005; Walzlein et al., 2008). However, fewer NPCs are observed migrating toward tumors of older mice (Walzlein et al., 2008), which are larger than tumors of young mice. Chirasani et al. (2010) showed *in vitro*, that NPC-derived bone morphogenetic protein 7 (Bmp 7) acts as a tumor suppressor, decreasing the proliferation, self-renewal and tumor-initiation ability of GBM stem cells (Chirasani et al., 2010).

Surprisingly, it has also been shown that neural progenitor cells are able to reach the tumor mass when injected systemically. Neural stem cells were shown to transmigrate across the BBB and reach intracranial gliomas (Díaz-Coránguez et al., 2013). Apparently, trans-endothelial migration of NPCs is mediated by CD44 (the same molecule used by activated leucocytes during diapedesis) and can thus be blocked by soluble hyaluronic acid (its ligand) or anti-CD44 blocking antibodies (Rampon et al., 2008). Hepatocyte growth factor (HGF), VEGF, PGE_2_ among other factors present in the conditioned medium of C6 glioma cells, were also found to induce NPC transmigration (Díaz-Coránguez et al., 2013). This ability to cross the barrier created by endothelial cells and astrocytes and to reach the brain parenchyma is being exploited as a possible therapeutic approach for many CNS diseases, including GBM.

## Exosomes, extracellular miRNAs and the blood-brain barrier

Naturally existing extracellular vesicles have been exploited as carriers for therapeutics to the CNS. Exosomes represent a class of these vesicles, and emerging evidence describes them as vehicles that have the ability to cross the BBB. Exosomes are small cup-shaped extracellular vesicles with diameters ranging from 30 to 100 nm. Exosomes are originated from the endocytic pathway, generated upon fusion of multivesicular endosomes (MVBs) to the plasma membrane and the consequent release of intraluminal vesicles (ILVs) of MVBs into the extracellular environment. At this point, ILVs are referred to as exosomes (Simons and Raposo, 2009; Raposo and Stoorvogel, 2013). The potential of exosomes to cross the BBB and dispatch molecules to the brain was first demonstrated by Alvarez-Erviti et al. (2011), who designed therapeutic exosomes and delivered them into the mouse brain (Alvarez-Erviti et al., 2011). Cells from bone marrow of C57BL/6 mice were harvested and stimulated with interleukins to produce immature dendritic cells, which were engineered to express Lamp2b, an exosomal membrane resident protein, fused with the rabies virus glycoprotein (RVG), which is internalized by brain cells upon specific interaction with the acetylcholine receptor. Purified exosomes released by dendritic cells were loaded with exogenous GAPDH siRNA or BACE 1 siRNA by electroporation, and systemically injected into mice in an allogeneic way. Specific gene knockdown was observed in neurons, microglia and oligodendrocytes, suggesting efficient exosome uptake by these cells. In another report, exosomes were used to entrap anti-inflammatory drugs, which were then delivered to the mouse brain by intranasal administration (Zhuang et al., 2011). The anti-inflammatory agents used were curcumin, a polyphenol compound that exhibits anti-inflammatory, antineoplastic and antioxidant activity; and JSI-124, a Stat3—Signal transducer and activator for transcripton 3—inhibitor. The drugs were loaded into exosomes and used in three different approaches: an LPS-induced brain inflammation model, experimental autoimmune encephalomyelitis (EAE) disease and mice bearing intracerebral GL26 tumor cells. The loaded exosomes rapidly reached the brain and were taken up by microglial cells. Curcumin exosomes led to induction of apoptosis of inflammatory microglia cells in the LPS-induced brain inflammation model. Exosomal curcumin treatment also delayed and attenuated EAE disease. JSI-124-containing exosomes significantly reduced brain tumor growth in the GL26 tumor model. Taken together, these findings suggest the possibility of using exosomes as drug carriers across biological barriers and as a promising non-immunogenic means to deliver therapeutics to the brain.

As cited above, exosomes could be used as vehicle to selectively deliver therapeutic nucleic acid based or conventional drugs, especially in those tumors that are difficult to access with chemotherapeutics due to the BBB (Ohno et al., 2013; Sun and Liu, 2014). For example, there is a number of *in vitro* and *in vivo* microRNAs (miRNAs) that had been mentioned in the context of altered signaling pathways, whose expression could represent potential therapeutic targets in GBM (Sana et al., 2011). The ability of individual miRNAs to target multiple genes and pathways could be a major advantage in GBM treatment (Purow, 2011). One of the most critical issues though, is how to deliver the agent (a miRNA mimic or inhibitor) to regions protected by the BBB (van Rooij et al., 2008; Gomes-da-Silva et al., 2013; Mizoguchi et al., 2013).

Recently, remarkable attention has been paid to the potential of circulating miRNAs profiling for cancer diagnosis and prognosis (Kosaka et al., 2010; Fujita et al., 2014). A pilot study demonstrated that miR-15b and miR-21 assessed in CSF were good markers for gliomas (Baraniskin et al., 2012). Glioblastoma tumor cells release exosomes containing miRNAs, messenger RNAs and angiogenic proteins (Skog et al., 2008; van der Vos et al., 2011; Yang et al., 2013; Figure 2A). microRNAs are molecules involved in gene regulation through a mechanism of inhibition or degradation of mRNAs of several known targets (Zhang et al., 2012). They can be detected in CSF, serum and plasma (Alexandrov et al., 2012; Teplyuk et al., 2012; Ilhan-Mutlu et al., 2013; Jin et al., 2013; Figure 2). Extracellular miRNAs are released from cells enclosed within exosomes, microvesicles, apoptotic bodies or in protein-miRNA complexes, such as high-density lipoprotein (HDL) and AGO2, the key effector protein of miRNA-mediated silencing (Valadi et al., 2007; Zernecke et al., 2009; Arroyo et al., 2011; Kosaka and Ochiya, 2011; Vickers et al., 2011). microRNAs detected in CSF of brain-cancer patients may be derived from brain-cancer cells; from surrounding brain tissues; or from extra-cranial tissue, as a consequence of BBB disruption and cancer treatments (Teplyuk et al., 2012). Glioblastoma derived microvesicles are likely to represent one of the mechanisms by which tumor cells change the brain microenvironment and make it more permissive for growth and invasion. As such one cannot disregard them as a powerful, non-invasive approach for diagnosing tumor progression in cancer patients (Skog et al., 2008).

Despite the large amount of studies mentioning the importance of circulating miRNAs in the diagnosis and prognosis of diseases, the exact mechanism that determines or identifies the circulating miRNAs, and their biological function remains uncertain. Two theories regarding the export and the function of circulating miRNA have been suggested. Some researches defend that circulating miRNA can be key mediators of various cell-cell communication processes, while others support the idea that all types of circulating miRNA in the biological fluids can be merely by products of cellular activity and cell death (Turchinovich et al., 2012; Turchinovich and Cho, 2014). Kirschner et al. (2013) highlighted their concern about the increasing number of reports revealing the clinical potential of using miRNA levels in body fluids as potential biomarkers. According to these authors, the field of cell-free miRNA research is still in its infancy, and it has failed to adopt a set of standardized criteria for reporting the methodology used in the quantification of miRNAs. The delivery of miRNA and the ability to target specific tissues or cells while avoiding nonspecific delivery remains challenging. For this reason, RNA carriers, such as liposomes or exosomes, are under investigation as a possible source of effective delivery strategies approaches (Santos et al., 2010; Ohno et al., 2013; Figure 2B). Either way, future research is needed to improve drug delivery to brain tumors (Ningaraj et al., 2007).

## Clinical problems related to glioma tumors

### Corticoids and morbi-mortality in glioma patients

One of the main causes of morbidity and mortality in glioma patients is the induction of severe cerebral edema, which leads to brain herniation in up to 60% of patients with GBM (Silbergeld et al., 1991). Cerebral edema, the abnormal accumulation of water inside the brain parenchyma, is commonly seen in GBM patients (Figure 3). Peri-tumoral edematous fluid can accumulate in amounts up to 90 ml per day in severe cases (Ito et al., 1990). Within the rigid skull, rapid augmentation of brain volume leads to a sharp increase in ICP, which can result in decreased cerebral blood flow, ischemia, brain herniation and death (Papadopoulos et al., 2004).

Brain tumor-related edema (BTRE) is caused by two main mechanisms, vasogenic and cytotoxic. While BTRE is classically associated with vasogenic edema, cytotoxic edema is being increasingly implicated in the pathophysiology of peri-tumoral swelling (Ito et al., 1990). Vasogenic edema is caused by the disruption of the BBB, which allows leakage of fluids from the blood into the brain parenchyma (Ryan et al., 2012). It is hypothesized that the disturbance of the BBB is due to two main mechanisms: (1) decreased expression of functioning tight-junctions and disruption of normally expressed tight-junctions; and (2) increased endothelial pinocytosis and endothelial fenestrations (Kröll et al., 2009). Cytotoxic edema, on the other hand, is associated with glioma-induced neuronal cell death and neurodegeneration, leading to further brain swelling and neurological deficits (Savaskan et al., 2008).

Corticosteroids have been used for more than 50 years in the treatment of BTRE, and have revolutionized the care of brain-tumor patients (Galicich et al., 1961). Approximately 70% of glioma patients receive dexamethasone as an adjuvant treatment, with a significant decrease in deaths (Hempen et al., 2002). In humans, brain imaging studies with MRI and PET have shown decreased peritumor water content and reduced tumor capillary permeability after dexamethasone administration (Jarden et al., 1989; Sinha et al., 2004). However, although corticosteroids have been used for a long time, the mechanisms through which they reduce brain edema are still poorly understood. It has been suggested that corticosteroids reduce BTRE by decreasing the permeability of the tumor capillaries (Hedley-Whyte and Hsu, 1986; Heiss et al., 1996). The capillary permeability-reducing effect is partly explained by upregulation of the tight-junction protein occluding (Gu et al., 2009). Dexamethasone also modulates the expression of VEGF in brain-tumor cells as well as in BBB cells (Kim et al., 2008). *In vitro* experiments have shown reduced levels of VEGF and increased concentration of claudin in response to corticosteroids (Kröll et al., 2009). The corticosteroid-sparing effects of VEGF-inhibitors such as bevazicumab in GBM patients also provide indirect evidence of a VEGF-modulation mechanism of dexamethasone (Kreisl et al., 2009).

Other mechanisms implicated in steroid-reducing BTRE are reduced angiogenesis and diminished cytotoxicity, and the anti-inflammatory effect on reducing cytokine-induced BBB breakdown. Recent evidence has shown that administration of dexamethasone in a glioma-cell culture and an animal model reduced glioma-induced neuronal damage and normalized vessel morphology and vessel density in the peritumoral brain area (Fan et al., 2014). Other studies have shown a 50% reduction in lymphocyte and microglia peritumoral infiltration when animals are treated with corticosteroids (Badie et al., 2000). It is hypothesized that corticosteroid inhibition of NF-κβ causes reduction of edema via inhibition of cytokine-induced barrier breakdown and decreasing the expression of cell adhesion molecules, which mediate T-cell–BBB interactions and excessive leukocyte recruitment across the BBB (Pitzalis et al., 2002).

Given the adverse effects of corticosteroids, such as osteoporosis, myopathy and immunosuppression, corticosteroid-sparing agents are being developed. Corticorelin acetate, a corticotropin-releasing factor (CRF) analog, was studied in a phase I/II trial and seems to be well tolerated. Randomized phase III trials are now envisioned (Villalona-Calero et al., 1998). Not surprisingly, given the prominent role of VEGF in the pathophysiology of BTRE, VEGF antibodies (e.g., bevazicumab) and VEGF receptor inhibitors (e.g., cediranib, sorafenib, and sunitinib) are being studied (Gerstner et al., 2009). Bevazicumab, the best-studied agent so far, has already proven to have anti-edema properties and to have the potential to be a steroid-sparing agent (Vredenburgh et al., 2010). However, because of the increased risk of intra-cerebral hemorrhage and pulmonary thromboembolism, the use of bevazicumab is not yet recommended solely for the purpose of corticosteroid-sparing (Cohen et al., 2009).

Brain tumor-related edema is a life-threatening complication of gliomas, and so far, their treatment has relied on the use of corticosteroids. Steroids have pleiotropic effects on BTRE; however, due to their numerous side effects, corticosteroid-sparing agents are being developed as alternatives for BTRE treatment.

### Tumor-associated epileptic seizures

Epileptic seizures often occur in brain-tumor patients, with reports of seizure risk of 60–100% among low-grade tumors and 40–60% in GBM (Vecht et al., 2014). It is the presenting symptom in 30–50% of the patients, and 10–30% will continue to have recurrent seizures, which contribute to the morbidity of the disease. Clinically, tumor-related seizures manifest as simple or complex partial seizures, with or without secondary generalization. A recent study evaluating the seizure semiology and short-term developments of primary brain tumors, found that the initial seizures were tonic-clonic in 48% of the patients, focal motor in 26%, complex partial in 10% and somatosensitive in 8%. The majority of cases (60%) had isolated seizures or a low seizure frequency at the onset of the disease, whereas a high seizure frequency or status epilepticus was observed in 18% and 12% of the cases, respectively (Michelucci et al., 2013).

The incidence of seizures differs widely between low- and high-grade tumors, probably with different pathophysiological mechanisms. There is an inverse relationship between the rate of tumor growth and the risk of seizures, which are more prevalent in tumors with a slower growth rate (Schaller and Rüegg, 2003; Chang et al., 2008). Besides the growth rate, the tumor location is another important element for the development of seizures. In general, patients with seizures at the onset of disease have a more-cortical location and are among the patients with a better prognosis.

Studies suggest that specific symptoms of the disease reflect not only the location of the tumor but also its biological behavior, because patients with low-grade glioma with early seizures in the beginning of the disease and concomitant early control have a higher survival rate than those who develop recurrent seizures (Danfors et al., 2009).

The pathogenesis of tumor-related seizures is complex, multifactorial and still not fully understood. The mechanisms of epileptogenesis differ among tumor types. The intrinsic epileptogenicity of glioneuronal tumors is supported by electrocorticography and surgical and immunocytochemical studies, suggesting the presence of a hyperexcitable neuronal component (Ferrier et al., 2006). The role of specific neuronal populations in epileptic foci was studied by comparing epileptic and nonepileptic cortex removed from patients with low-grade gliomas. Epileptic and nearby (within 1 to 2 cm) nonepileptic temporal lobe neocortex was identified using electrocorticography. Cortical specimens taken from four patients identified as epileptic and nonepileptic were all void of tumor infiltration. Somatostatin- and γ-aminobutyric acid (GABAergic)-immunoreactive neurons were identified and counted. Although there was no significant difference in the overall cell count, the authors found a significant decrease in both somatostatin- and GABAergic-immunoreactive neurons (74% and 51%, respectively) in the epileptic cortex compared to that in the nonepileptic cortex from the same patient. These findings suggest that changes in neuronal subpopulations may have a role in the onset and propagation of epileptiform activity in patients with low-grade gliomas. Gliosis and chronic inflammatory changes in the perilesional regions of these tumors seem also to participate in the generation of seizures. Some other possible mechanisms of epileptogenesis in high-grade gliomas are the perilesional focal ischemia, deafferentation of cortical areas by mass effect, and also increased iron in minor bleeding (Beaumont and Whittle, 2000).

The seizures disrupt the BBB, enhance inflammatory responses, increase glutamate excitotoxicity and cell apoptosis, and substantially increase cognitive and psychological morbidity. Several studies have described variations in neurovascular integrity and disturbances of the BBB with neuronal hypersynchronization and epileptiform activity (Liebner et al., 2000). Molecular changes in brain tumors can affect the BBB structure and functions, including decreased expression of transmembrane junctional proteins. Disruption of the BBB may also lead to abnormal extravasation of plasma protein and other substances, including glutamate, contributing to hyperexcitability and development of seizure focus (Ivens et al., 2007).

The BBB has an important role in the immune response, with elevated production of inflammatory cytokines (Vezzani and Granata, 2005). The supraregulation of adhesion molecules and metalloproteinases contributes to the changes in the BBB permeability.

Because seizures substantially increase morbidity and some patients evolve with medically refractory epilepsy, it is important to understand the mechanism of action and the effect of the drug of choice for treating these patients. The recurrence or worsening of seizures is often associated with tumor recidivism in GBM, and they require additional pharmacological treatment, which can be hard to manage because of the many drug interactions and cumulative side effects.

The current consensus is that all patients with brain tumors presenting with or having seizures should be treated with antiepileptic drugs (AEDs), because of the high risk of recurrence of seizures. The first-line AEDs, including valproate (VPA), phenytoin and carbamazepine, have demonstrated many major side effects and dangerous interactions with chemotherapeutic and antitumor drugs. The most commonly seen side effects with these drugs are cognitive impairment, liver dysfunction, dermatological reactions and bone-marrow suppression. Several studies have found that side effects are more common in patients with brain tumors than in the general epileptic population.

Drug interactions between AEDs and commonly used tumor therapies can lead to inadequate control of the seizures or sub-therapeutic treatment of the tumor. Toxic effects have also been noted. Many of the common AEDs, especially carbamazepine, phenytoin and phenobarbital, induce cytochrome P450 enzymes, which accelerate the metabolism, reducing the half-life of corticosteroids and many other chemotherapy drugs that in this context exhibit decreased therapeutic effectiveness. Some chemotherapeutic agents also induce the P450 system, lowering concentrations of the AED.

Among the older AEDs, valproate, in doses of 1–3 g per day, has been claimed to be the drug of choice because of its anticonvulsive efficacy. It has an intravenous formulation, which is very useful in perioperative management and also in cases of status epilepticus, which is seen in up to 26% of these patients, with the overall mortality rate reaching 30–40%. Also, its action as a histone deacetylase inhibitor may reduce proliferation rates and promote differentiation of cancer stem cells, the main mechanism responsible for tumor recidivism in the case of GBM (Alvarez et al., 2014). Valproate may increase the hematological toxicity of the chemotherapy and may independently impair hemostasis, which is of some concern for these patients, who will often require surgical procedures.

Within the past 10 years, several new AEDs including lamotrigine, oxcarbazepine, topiramate, levetiracetam (LEV), lacosamide and zonizamide have emerged that are without clinically important drug interactions. Most of them have shown similarly good efficacy, with better tolerability for oxcarbazepine, lacosamide and lamotrigine. Most data were based on uncontrolled retrospective studies. Of this group, only levetiracetam and lacosamide have intravenous formulations.

Many studies have now proposed levetiracetam as the drug of choice in the treatment and prevention of peri- and post-operative seizures, in daily doses of 1.5–3 or 4 g. Levetiracetam has the advantages of excellent bioavailability (100%, equivalent to the venous formulation), no protein binding, no liver-metabolism or enzyme-inducer effect, and a very good tolerability profile. It has a completely different mechanism of action, binding to the Synaptic Vesicle Protein 2 A, thus inhibiting neurotransmitter release. It also has shown histone deacetylase inhibitor, anti-inflammatory and antioxidant effects. Many recent trials have demonstrated its anticonvulsive efficacy and safety in the control of perioperative (88 to 100% seizure control) and long-term epileptic seizures (91% seizure freedom), as well as in the treatment of status epilepticus when combined with phenytoin (Claassen et al., 2003) or with phenytoin and pregabalin (trifecta trial with 70% success in halting the status epilepticus; Swisher et al., 2012).

In a retrospective comparative study between valproate and levetiracetam with 282 patients with supratentorial brain tumors (Lee et al., 2013), the two drugs showed comparable efficacy (*ca*. 7% of patients experienced postoperative seizures) and much better tolerability for levetiracetam (long-term complication rates of 26.8% for VPA and 9.8% for LEV). Moreover, 38.5% in the VPA group changed to or added another AED, whereas only 17.6% in the LEV group did so. Another retrospective comparative study (Kerkhof et al., 2013) with 291 GBM patients treated with VPA alone, with LEV alone or with a combination of the two as a second option in the refractory group, also showed a similar efficacy in seizure control (seizure freedom of 78% for VPA and 70% for LEV), although VPA was slightly superior with respect to the overall long-term survival rate, with a 2-month-longer survival. The polytherapy with VPA and LEV combined added 60% seizure freedom in both refractory monotherapy groups.

The clinical management of seizures in this context is complex, requiring experts in this field, measurement of serum levels of the antiepileptic and oncologic drugs, serial blood analysis, monitoring of clinical side effects, and keeping in mind that the recurrence of seizures may represent a tumor recurrence.

## Conclusion and perspectives

In this review, we have pointed out and discussed the implications of the interactions between normal glia or glioma cells and the BBB. Since its first discovery by Edwin E. Goldman, considerable progress has been made in the understanding of BBB. Although 100 years have passed since the BBB discovery, the mechanisms by which some drugs or parasites enter the brain are still unsolved. Furthermore, the real interactions of the endothelial cells with the extra-endothelial components of the brain microenvironment remain unclear. It is not completely understood how astrocytes endfeet interact with BBB and the astrocytoma derived signals that lead to an intense vasculogenesis into the tumor. Interestingly, GBM and BBB interactions may occur via extracellular proteins. For instance, the imbalance of tenascin and fibronectin in the tumor contributes to vessel formation as we have previously demonstrated (Alves et al., 2011a). Glioblastoma, a dramatic and fatal tumor that grows into the brain, can create a scenario of seizures very difficult to control. The growth of this tumor can also promote disruption of vessels, generating edema, which complicates the health status of the patient. It is clear that novel methods to control GBM growth and BBB disruption are necessary to solve these severe complications.

## Conflict of interest statement

The authors declare that the research was conducted in the absence of any commercial or financial relationships that could be construed as a potential conflict of interest.
